# A Novel Homozygous Non-sense Mutation in the Catalytic Domain of MTHFR Causes Severe 5,10-Methylenetetrahydrofolate Reductase Deficiency

**DOI:** 10.3389/fneur.2019.00411

**Published:** 2019-04-24

**Authors:** Salam Massadeh, Muhammad Umair, Manal Alaamery, Majid Alfadhel

**Affiliations:** ^1^Developmental Medicine Department, King Abdullah International Medical Research Center, King Saud Bin Abdulaziz University for Health Sciences, King Abdulaziz Medical City, Ministry of National Guard Health Affairs, Riyadh, Saudi Arabia; ^2^Joint Centers of Excellence Program, KACST-BWH/Harvard Center of Excellence for Biomedicine, King Abdulaziz City for Science and Technology (KACST), Riyadh, Saudi Arabia; ^3^Medical Genomics Research Department, King Abdullah International Medical Research Center (KAIMRC), King Saud Bin Abdulaziz University for Health Sciences, Ministry of National Guard Health Affairs, Riyadh, Saudi Arabia; ^4^Division of Genetics, Department of Pediatrics, King Abdullah Specialized Children's Hospital, King Abdulaziz Medical City, Riyadh, Saudi Arabia

**Keywords:** *MTHFR*, non-sense mutation, white matter disease, microcephaly, severe methylenetetrahydrofolate reductase deficiency

## Abstract

**Background:** Severe 5,10-methylenetetrahydrofolate reductase (MTHFR) deficiency is a heterogeneous metabolic disorder inherited in an autosomal recessive manner. Pathogenic mutations in *MTHFR* gene have been associated with severe MTHFR deficiency. The clinical presentation of MTHFR deficiency is highly variable and associated with several neurological anomalies.

**Methods:** Direct whole-exome sequencing (WES) was performed in all the five available individuals from the family, including the affected individual (III-7) using standard procedures.

**Results:** We observed a proband (III-7) with an abnormality in the cerebral white matter, apnoea, and microcephaly. WES analysis identified a novel homozygous non-sense mutation (c.154C>T; p.Arg52^*^) in *MTHFR* gene that segregated with the disease phenotype within the family.

**Conclusion:** We identified a novel non-sense mutation in *MTHFR* gene in a single Egyptian family with severe MTHFR deficiency. The present investigation is clinically important, as it adds to the growing list of *MTHFR* mutations, which might help in genetic counseling of families of affected children and proper genotype-phenotype correlation.

## Background

Severe 5,10-Methylenetetrahydrofolate reductase (MTHFR; OMIM 236250) deficiency is a rare inborn error of metabolism and inherited in an autosomal recessive fashion. It is a very common disorder of folate metabolism and is clinically characterized with low plasma methionine level, high homocysteine level, homocystinuria, and hyperhomocysteinemia (1, 2).

*MTHFR* gene is responsible for the assembly of MTHFR enzyme, which plays a very important role in the catalysis of 5,10-methyltetrahydrofolate (THF) to 5-methyltetrahydrofolate (an NADPH-dependent irreversible reduction) to obtain methionine from homocysteine (Supplementary Figure 1). Methyl THF is the most common form of folate in tissues and the plasma and serves as the methyl group donor in the methyl THF-homocysteine S-methyltransferase reaction involving remethylation of homocysteine to methionine (3).

The deficiency of MTHFR enzyme causes an increase in the concentration of homocysteine and methionine, resulting in severe neurological phenotypes. Patients having neonatal onset (i.e., early onset <1-year-old) MTHFR deficiency are mostly associated with feeding problems, neurological phenotype, microcephaly, seizures, and communicating hydrocephalus, while the late onset is characterized with delayed developmental milestones, cognitive impairment and/or gait abnormalities, stroke, apnoea, paresthesia, psychiatric disturbances, and neurological deterioration, which lead to coma and even death (4, 5).

In the present study, we performed direct whole exome sequencing (WES) to ascertain the genetic cause of severe MTHFR deficiency in the single affected individual (proband). As a result, we identified a homozygous non-sense mutation in *MTHFR* gene located on chromosome 1p36.22.

## Case Presentation

### Clinical Examination

**Figure 1:**
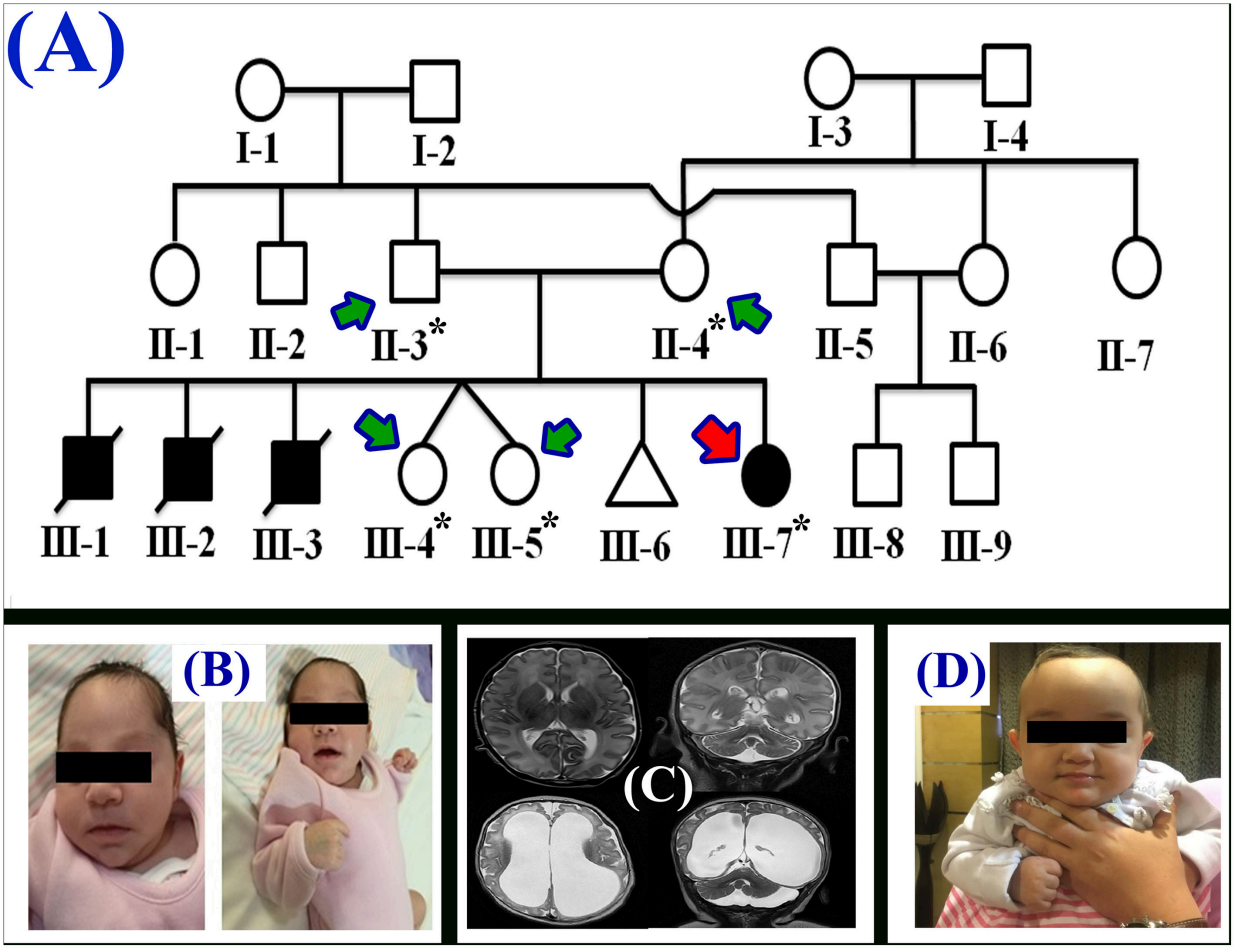
**(A)** Pedigree of the family segregating severe MTHFR deficiency. Pedigree clearly depicts an autosomal recessive mode of inheritance. Circles and squares represent females and males, respectively. Clear symbols represent normal, while filled symbols represent affected individuals. Slashes represents deceased individuals and triangle represents spontaneous abortion (SAB). The individual numbers labeled with asterisks were subjected to WES and the red arrow indicates the proband. **(B)** Pictures of the affected individual exhibiting microcephalic features at 1 month of age. **(C)** Brain MRI showing axial, coronal, and both T2 weighted images. These images revealed bilateral abnormal and symmetrical high signal intensity along the bilateral frontal horn in white matter, and the superior part of the periventricular deep white matter as well as within the subcortical basal temporal lobe white matter. The images also showed severely dilated ventricles and loss of white matter in cerebral hemispheres with prominent extra-axial CSF spaces. **(D)** A recent photograph of the proband (III-7) showing severe MTHFR deficiency.

The proband (III-7) genetically and clinically analyzed in the present study belongs to a non-consanguineous Egyptian family (Figure 1A). The parents had total seven children, three had deceased (III-1, III-2, and III-3) and showed (i.e., early onset <1 year old) similar phenotypes as the proband, healthy twin daughters (III-4 and III-5), one miscarriage (III-6), and the proband (III-7) (early onset <1 year). The deceased children age were: III-1:40 days, III-2: 2 months, III-3: 45 days; and all the deceased children became lethargic, hypoactive with poor suckling, with associated prolonged sleepiness. These were referred to the neonatal intensive care unit (NICU) as quarry neonatal sepsis. Computed tomography (CT) scan revealed subarachnoid hemorrhage and the patients died of apnoea, disseminated intravascular coagulation (DIC), and cardiorespiratory arrest.

### First Clinical Evaluation

During the first clinical investigation, the proband (female; III-7) was 1-month-old with features such as non-obstructive hydrocephalus, recurrent apnea, microcephaly, and white matter abnormality (Figure 1B). The proband showed delayed growth during the last month of gestation. At birth, the head circumference was 31.5 cm, and the birth weight was 2,980 grams (Figure 1B). After 14 days, the proband developed apnea attacks and bradypnea and showed difficulties with swallowing. Ophthalmological examination demonstrated bilateral optic nerve hypoplasia, left retinal multiple hemorrhages, and normal anterior segment. The laboratory screening results revealed low white blood cell count, low platelet count, and high number of lymphocytes. Following multiple episodes of apnea, multi-sequential and multiplanar magnetic resonance imaging (MRI) was performed. The MRI findings showed bilateral abnormal almost symmetrical T2. High signal intensity was observed in the white matter along the bilateral horn deep in the white matter, suggestive of white matter disease (Figure 1C). No chest infection, deafness, or cardiovascular anomaly was noted. The proband had a total blood homocysteine level of 163 nmol/mL (normal, 3.7–12.5 nmol/mL) and the plasma methionine range of < 5 (10–39 μmol/L). The proband was on regular medication such as betaine (100 mg·kg^−1^·day^−1^), methionine (50 mg·kg^−1^·day^−1^), vitamin B12 (5 mg PO 1/day), and L-5-MTHF (15 mg PO 1/day).

### Follow-Up Examination

Follow-up examination after 10 months revealed that the proband could support her head but could not sit without support and may easily roll over (Figure 1D). Ophthalmic examination revealed optic atrophy, retinal hemorrhage, and the affected individual was unable to fix eyes nor follow. No hearing abnormality was observed. The proband was able to make sounds at the age of 4–5 months. Physical examination revealed a height of 79 cm (>95th percentile), weight of 10.7 kg (>95th percentile), and head circumference of 48 cm (>95th percentile). The total blood homocysteine level was 68 nmol/mL (normal, 3.7–12.5 nmol/mL), while the plasma methionine level was 54 μmol/L (normal, 10–39 μmol/L).

## Materials and Methods

### Study Approval and Sample Collection

The present study was approved by the Institutional Review Board (KAIMRC) and followed Helsinki protocols. Written informed consent was obtained from the parents of the participant for the research study, presentation of photographs/radiographs, and publication of this case report.

### DNA Extraction

DNA was extracted from peripheral blood samples using QIAamp DNA Micro kit (Hilden, Germany) and quantified using NanoDrop™ spectrophotometer using standard procedures.

### WES

WES was commercially performed (Centogene, Rostock, Germany) for five family members, including the single affected individual (proband: III-7), both parents (II-3 and II-4), and twin sisters (III-4 and III-5) using Illumina platform (Illumina, Inc., San Diego, CA, USA; Figure 1A). RNA capture baits against approximately 60 Mb of the human exome (targeting >99% of regions in the consensus coding sequence, RefSeq, and Gencode databases) were used to enrich the regions of interest from the fragmented genomic DNA with the SureSelect Human All Exon V6 kit (Agilent Technologies, Santa Clara, CA, USA). The generated library was sequenced to obtain an average coverage depth of about 100X. In general, about 97% of the targeted bases are covered in more than 10X. Data analysis and interpretation were performed by Centogene using an end-to-end in-house bioinformatic pipeline with applications including base calling and alignment of reads to the GRCh37/hg19 (GRCh37; http://genome.ucsc.edu/) genome assembly.

### Filtering of the Disease-Causing Variants

Primary filtering out of low-quality reads and probable artifacts and the subsequent annotation of variants were performed using standard methods. As the pedigree depicted autosomal recessive inheritance, homozygous and compound heterozygous variants were preferred but heterozygous variants were not ignored.

The variants were filtered and validated using standard methods (6) and screened in different databases such as dbSNP (https://www.ncbi.nlm.nih.gov/projects/SNP/), 1000 genomes Project (http://www.internationalgenome.org/), ExAC (http://exac.broadinstitute.org/), and gnomAD (http://gnomad.broadinstitute.org/). Screening for the disease-causing variants (homozygous and compound heterozygous) led to the identification of a novel homozygous non-sense mutation (c.154C>T; p.Arg52^*^) in the exon 1 of *MTHFR* gene (NM_005957.4). Both the parents were heterozygous for the identified variant. The identified variant was absent in different available databases (dbSNP, 1000 genomes, ExAC, and gnomAD) and several in-house exomes (control).

### *In silico* Analysis

Pathogenicity of the identified variant was evaluated with different online mutation prediction tools such as MutationTaster (mutationtaster.org), DNN (https://cbcl.ics.uci.edu/public_data/DANN/), FATHMM (fathmm.biocompute.org.uk), LRT (genetics.wustl.edu/jflab/lrt_query.html), VarSome (https://varsome.com/), CADD—Combined Annotation Dependent Depletion (https://cadd.gs.washington.edu/), and MutationAssessor (mutationassessor.org).

The identified variant (c.154C>T) was also subjected to Sanger sequencing with all the available members of the family using standard methods (Centogene, Germany), and the variant was shown to perfectly segregate with the disease phenotype.

## Discussion and Conclusions

MTHFR (EC 1.5.1.20) is a key enzyme of the folate cycle involved in the catalysis of 5,10-methylenetetrahydrofolate to 5-methyltetrahydrofolate, which is required for the remethylation of homocysteine to methionine. Methionine is subsequently metabolized to form S-adenosylmethionine. Thus, any disturbance in the pathway, either through the supply of cobalamin/folate or MTHFR deficiency, may cause accumulation of homocysteine, leading to altered patterns of methylation [(7–9); Supplementary Figure 1].

In the present study, we investigated a single affected individual (III-7) with MTHFR deficiency demonstrating severe features such as microcephaly, delayed growth, ophthalmic issues, non-obstructive hydrocephalus, elevated blood homocysteine level, and elevated plasma methionine levels. Features reported in the proband were similar to the previously reported cases (9–13). The affected individual had an early onset (<1 year) of the disease. It has been previously observed that the patients with early onset exhibit lesser residual enzyme activity than those with late onset (>1 year) (12). Residual enzyme activity was not tested in the present study. We performed WES for all the available members of the family (Figure 1A) and identified a homozygous non-sense mutation (c.154C>T; p.Arg52^*^) in *MTHFR* gene. The non-sense mutation (p.Arg52^*^) was located within the catalytic domain and most likely resulted in the complete loss of function of the MTHFR protein, either through non-sense-mediated mRNA decay or via the production of a truncated MTHFR protein.

**Figure 2:**
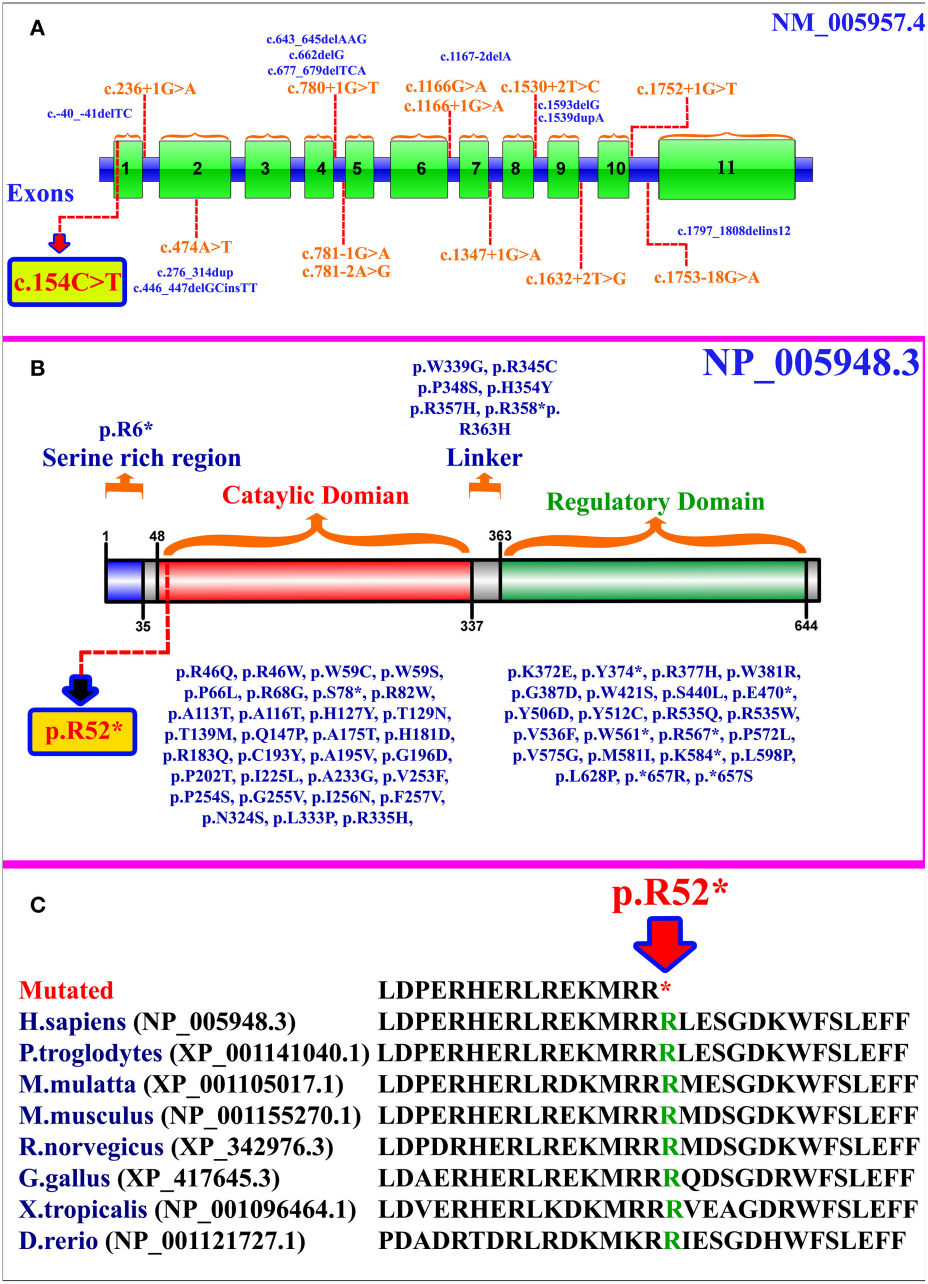
**(A)** *MTHFR* gene structure highlighting the already reported splice site and small deletion mutations. The variant identified in the present study is represented within a box. **(B)** Schematic representation of MTHFR protein and different domains. Missense and non-sense mutations are shown along each domain. The mutation identified in the present study is represented within a box. **(C)** Partial amino acid sequence of MTHFR protein showing conservation of Arg52 amino acid residue across different species.

*MTHFR* gene is located on chromosome 1p36.22, exhibits 11 exons, and encodes a 656 amino acid protein (NP_005948.3) (Figure 2A). MTHFR protein exists as a homodimer comprising two N-terminal catalytic domains with binding sites for NADPH, methylene-THF, and cofactor FAD-linked to the regulatory domain (C-terminus) (Figure 2B). The mutation (p.Arg52^*^) identified herein is highly conserved across different species (Figure 2C). The exact association between the position of mutation and clinical phenotypes is yet unknown, as a biallelic loss-of-function mutation in any part of the protein results in extreme enzyme deficiency. This phenomenon highlights the importance of the complete peptide for proper enzyme activity (14).

As per our knowledge, 132 mutations have been reported in *MTHFR* gene that may cause several disorders such as homocystinuria, anencephaly, tetralogy of Fallot with cleft lip/palate, MTHFR deficiency, acute aortic dissection, reduced enzyme activity, association with preeclampsia, increased erythrocyte folate, respiratory failure and hypotonia, coronary heart disease, Ehlers-Danlos syndrome IV, and Facial palsy (11, 13, 15–18). Clinical comparison of patients with non-sense mutations in *MTHFR* gene has been compiled in Supplementary Table 1. Only 84 different mutations have been described in the literature that cause severe MTHFR deficiency, including 52 missense mutations, 12 splice acceptor/donor site, 8 non-sense mutations, 7 small deletions, 2 small indels, 2 start loss, and 1 gross insertion (Table 1). The majority of the mutations causing severe MTHFR deficiency are reported in the catalytic domain (Figure 2B).

**Table 1:** Mutations reported in the *MTHFR* gene causing a severe 5-10-MTHFR deficiency.

	**Disorder**	**Amino acid change**	**Nucleotide change**	**Mutation type**	**Exon/Intron**	**Domain**
1	MTHFR deficiency	Arg6*	c.16A>T	Non-sense	Exon 1	Serine rich
2	MTHFR deficiency	Arg46Gln	c.137G>A	Missense	Exon 1	Catalytic
3	MTHFR deficiency	Arg46Trp	c.136C>T	Missense	Exon 1	Catalytic
4	MTHFR deficiency	Trp59Cys	c.177G>T	Missense	Exon 1	Catalytic
5	MTHFR deficiency	Trp59Ser	c.176G>C	Missense	Exon 1	Catalytic
6	MTHFR deficiency	Pro66Leu	c.197C>T	Missense	Exon 1	Catalytic
7	MTHFR deficiency	Arg68Gly	c.202C>G	Missense	Exon 1	Catalytic
8	MTHFR deficiency	Ser78*	c.233C>G	Non-sense	Exon 1	Catalytic
9	MTHFR deficiency	Arg82Trp	c.244C>T	Missense	Exon 2	Catalytic
10	MTHFR deficiency	Ala113Thr	c.337G>A	Missense	Exon 2	Catalytic
11	MTHFR deficiency	Ala116Thr	c.346G>A	Missense	Exon 2	Catalytic
12	MTHFR deficiency	His127Tyr	c.379C>T	Missense	Exon 2	Catalytic
13	MTHFR deficiency	Thr129Asn	c.386C>A	Missense	Exon 2	Catalytic
14	MTHFR deficiency	Thr139Met	c.416C>T	Missense	Exon 2	Catalytic
15	MTHFR deficiency	Gln147Pro	c.440A>C	Missense	Exon 2	Catalytic
16	MTHFR deficiency	Ala175Thr	c.523G>A	Missense	Exon 3	Catalytic
17	MTHFR deficiency	His181Asp	c.541C>G	Missense	Exon3	Catalytic
18	MTHFR deficiency	Arg183Gln	c.548G>A	Missense	Exon 3	Catalytic
19	MTHFR deficiency	Cys193Tyr	c.578G>A	Missense	Exon 3	Catalytic
20	MTHFR deficiency	Ala195Val	c.584C>T	Missense	Exon 3	Catalytic
21	MTHFR deficiency	Gly196Asp	c.587G>A	Missense	Exon 3	Catalytic
22	MTHFR deficiency	Pro202Thr	c.604C>A	Missense	Exon 4	Catalytic
23	MTHFR deficiency	Ile225Leu	c.673A>C	Missense	Exon 4	Catalytic
24	MTHFR deficiency	Ala233Gly	c.698C>G	Missense	Exon 4	Catalytic
25	MTHFR deficiency	Val253Phe	c.757G>T	Missense	Exon 4	Catalytic
26	MTHFR deficiency	Pro254Ser	c.760C>T	Missense	Exon 4	Catalytic
27	MTHFR deficiency	Gly255Val	c.764G>T	Missense	Exon 4	Catalytic
28	MTHFR deficiency	Ile256Asn	c.767T>A	Missense	Exon 4	Catalytic
29	MTHFR deficiency	Phe257Val	c.769T>G	Missense	Exon 4	Catalytic
30	MTHFR deficiency	Asn324Ser	c.971A>G	Missense	Exon 5	Catalytic
31	MTHFR deficiency	Leu333Pro	c.998T>C	Missense	Exon 5	Catalytic
32	MTHFR deficiency	Arg335His	c.1004G>A	Missense	Exon 5	Catalytic
33	MTHFR deficiency	Trp339Gly	c.1015T>G	Missense	Exon 5	Catalytic
34	MTHFR deficiency	Arg345Cys	c.1033C>T	Missense	Exon 5	Catalytic
35	MTHFR deficiency	Pro348Ser	c.1042C>T	Missense	Exon 6	Regulatory
36	MTHFR deficiency	His354Tyr	c.1060C>T	Missense	Exon 6	Regulatory
37	MTHFR deficiency	Arg357His	c.1070G>A	Missense	Exon 6	Regulatory
38	MTHFR deficiency	Arg358*	c.1072C>T	Non-sense	Exon 6	Regulatory
39	MTHFR deficiency	Arg363His	c.1088G>A	Missense	Exon 6	Regulatory
40	MTHFR deficiency	Lys372Glu	c.1114A>G	Missense	Exon 6	Regulatory
41	MTHFR deficiency	Tyr374*	c.1122C>G	Non-sense	Exon 6	Regulatory
42	MTHFR deficiency	Arg377His	c.1130G>A	Missense	Exon 6	Regulatory
43	MTHFR deficiency	Trp381Arg	c.1141T>C	Missense	Exon 6	Regulatory
44	MTHFR deficiency	Gly387Asp	c.1160G>A	Missense	Exon 6	Regulatory
45	MTHFR deficiency	Trp421Ser	c.1262G>C	Missense	Exon 7	Regulatory
46	MTHFR deficiency	Ser440Leu	c.1319C>T	Missense	Exon 7	Regulatory
47	MTHFR deficiency	Glu470*	c.1408G>T	Non-sense	Exon 8	Regulatory
48	MTHFR deficiency	Tyr506Asp	c.1516T>G	Missense	Exon 8	Regulatory
49	MTHFR deficiency	Tyr512Cys	c.1535A>G	Missense	Exon 9	Regulatory
50	MTHFR deficiency	Arg535Gln	c.1604G>A	Missense	Exon 9	Regulatory
51	MTHFR deficiency	Arg535Trp	c.1603C>T	Missense	Exon 9	Regulatory
52	MTHFR deficiency	Val536Phe	c.1606G>T	Missense	Exon 9	Regulatory
53	MTHFR deficiency	Trp561*	c.1683G>A	Non-sense	Exon 10	Regulatory
54	MTHFR deficiency	Arg567*	c.1699C>T	Non-sense	Exon 10	Regulatory
55	MTHFR deficiency	Pro572Leu	c.1715C>T	Missense	Exon 10	Regulatory
56	MTHFR deficiency	Val575Gly	c.1724T>G	Missense	Exon 10	Regulatory
57	MTHFR deficiency	Met581Ile	c.1743G>A	Missense	Exon 10	Regulatory
58	MTHFR deficiency	Lys584*	c.1750A>T	Non-sense	Exon 10	Regulatory
59	MTHFR deficiency	Leu598Pro	c.1793T>C	Missense	Exon 11	Regulatory
60	MTHFR deficiency	Leu628Pro	c.1883T>C	Missense	Exon 11	Regulatory
61	MTHFR deficiency	*657Arg	c.1969T>C	Stop loss	Exon 11	Regulatory
62	MTHFR deficiency	*657Ser	c.1970G>C	Stop loss	Exon 11	Regulatory
63	MTHFR deficiency	–	c.236+1G>A	Splice	Intron 2	Catalytic
64	MTHFR deficiency	–	c.474A>T	Splice	Exon 2	Catalytic
65	MTHFR deficiency	–	c.781-2A>G	Splice	Intron 5	Catalytic
66	MTHFR deficiency	–	c.781-1G>A	Splice	Intron 5	Catalytic
67	MTHFR deficiency	–	c.780+1G>T	Splice	Intron 5	Catalytic
68	MTHFR deficiency	–	c.1166G>A	Splice	Exon 6	Catalytic
69	MTHFR deficiency	–	c.1166+1G>A	Splice	Intron 7	Catalytic
70	MTHFR deficiency	–	c.1347+1G>A	Splice	Intron 8	Regulatory
71	MTHFR deficiency	–	c.1530+2T>C	Splice	Intron 9	Regulatory
72	MTHFR deficiency	–	c.1632+2T>G	Splice	Intron 10	Regulatory
73	MTHFR deficiency	–	c.1753-18G>A	Splice	Intron 10	Regulatory
74	MTHFR deficiency	–	c.1752+1G>T	Splice	Intron 11	Regulatory
75	MTHFR deficiency	–	c.643_645delAAG	Small deletions	Exon 4	
76	MTHFR deficiency	–	c.-40_-41delTC	Small deletions 5′UTR	Intron 1	Serine rich
77	MTHFR deficiency	–	c.662delG	Small deletions	Exon 4	Catalytic
78	MTHFR deficiency	–	c.677_679delTCA	Small deletions	Exon 4	Catalytic
79	MTHFR deficiency	–	c.1167-2delA	Small deletions	Exon 7	Regulatory
80	MTHFR deficiency	–	c.1593delG	Small deletions	Exon 9	Regulatory
81	MTHFR deficiency	–	c.1539dupA	Small deletions	Exon 9	Regulatory
82	MTHFR deficiency	–	c.446_447delGCinsTT	Small indels	Exon 2	Catalytic
83	MTHFR deficiency	–	c.1797_1808delins12	Small indels	Exon 11	Regulatory
84	MTHFR deficiency	Leu89_Pro101dup	c.276_314dup	Gross insertions	Exon 2	Catalytic

Furthermore, *MTHFR* knockout mice showed a decrease in methionine level, methionine/homocysteine ratio, dimethylglycine level, and lipid deposition in proximal aorta along with changes in cerebellar pathology, reduced cerebellum, reduced cerebral cortex, and developmental issues (19–21). A therapeutic strategy employing biochemical methods is important to normalize methionine level and decrease the plasma levels of homocysteine. Treatment for severe MTHFR deficiency is heterogeneous, and different combination and dosage of drugs are prescribed depending on the severity of the disorder. High doses of betaine have been suggested for patients with severe MTHFR deficiency (22, 23). Vitamins B6 and B12 supplementation with folic acid is also significant in the conversion of homocysteine into methionine. In the present study, the proband was prescribed a combination of betaine, methionine, vitamin B12, and L-5-MTHF that improved the overall condition, as she became more active, alert, and oriented and survived as compared to her affected siblings. At present, the proband is 14 months old, shows no apnea or seizure; hydrocephalus is not progressive and developmentally she can roll over. However, she is unable to sit, crawl, or walk and functions as a 4–5 months old infant. Folic acid dosages in combination with folate/folinic acid, hydroxocobalamin, cyanocobalamin, riboflavin, and carnitine have also been previously prescribed to some patients that could partially reduce the clinical symptoms and biochemical abnormalities (5).

In conclusion, we identified a novel biallelic non-sense mutation in a single affected individual suffering from severe MTHFR deficiency. MTHFR deficiency is potentially treatable when properly diagnosed at early stages. Clinicians should be aware of the phenotypes related to early-late onset of the disease. Timely molecular diagnosis and treatment with high dose of methyl donors is critical for the survival of these patients. Identification of the pathogenic variants of *MTHFR* gene may extend the knowledge about the spectrum of mutations and highlight the proper genotype-phenotype correlation.

## Ethics Statement

The present study was approved by the Institutional Review Board (KAIMRC) and followed Helsinki protocols. Written informed consent was obtained from the parents of the participant for the research study, presentation of photographs/radiographs, and publication of this case report.

## Author Contributions

SM and MU drafted the manuscript, collected samples, clinical data, analyzed data, and performed experiments. MAla analyzed genomic data. MAla and MAlf edited the manuscript, while MAlf supervised the study.

### Conflict of Interest Statement

The authors declare that the research was conducted in the absence of any commercial or financial relationships that could be construed as a potential conflict of interest.
