# Supplementary material for: A Novel Homozygous Non-sense Mutation in the Catalytic Domain of MTHFR Causes Severe 5,10-Methylenetetrahydrofolate Reductase Deficiency
Source: Front Neurol. 2019 Apr 24;10:411. doi: 10.3389/fneur.2019.00411 (PMC6491806; doi:10.3389/fneur.2019.00411)
Supplement: Supplementary file 1 [file Data_Sheet_1.docx]

Supplementary Material

**Supplementary figure**

**Supplementary Figure 1:** Schematic representation of folate pathway and the effect of *MTHFR* gene mutation.

**Supplementary Table**

**Supplementary Table 1:** Clinical comparison of patients with a nonsense mutation in *MTHFR* gene, leading to severe methylenetetrahydrofolate reductase deficiency.

| **Families/patients with nonsense mutations** | **Clinical evaluations observed** |
| --- | --- |
| **Kluijtmans et al., 1998 (Patient K)** | Homocystinuria, internal hydrocephalus, severe psychomotoric retardation, severe muscular hypotonia, no social contact; plasma homocysteine (38–50 µm/L) and homocysteine–cysteine mixed disulfide (40–60 µm/L) levels were severely elevated, whereas plasma methionine concentration decreased |
| **Kluijtmans et al (1) (Patient U)** | Severely retarded psychomotor development, severe microcephaly, homocystinuria, methionine concentration in plasma (< 5 µm/L) and total plasma homocysteine (> 150 µm/L). Upon betaine treatment (6–9 g daily), plasma methionine level increased to normal values, and total plasma homocysteine level decreased to 90 µm/L |
| **Tonetti et al., (2) (Family 1)** | Hypotonia, hypothermia, neurological distress, respiratory failure, dilated cerebral ventricles, demyelinated white matter, and encephalopathy with seizures |
| **Tonetti et al., (2) (Family 5)** | Mental retardation, speech delay, delayed psychomotor development, mild cortical atrophy, and ventricular enlargement |
| **Al-Shamsi et al., (3)** | Progressive encephalopathy, seizure, cerebral venous thrombosis, gangrenous-like bullous formation in the leg, congenital heart disease, elevated homocysteine, decreased methionine level |
| **Present Study** | Severe neurological signs, recurrent apnea, microcephaly, white matter abnormality, ophthalmic issues, and elevated total blood homocysteine and plasma methionine levels |

**References for supplementary files:**

1. Kluijtmans LA, Wendel U, Stevens EM, van den Heuvel LP, Trijbels FJ, Blom HJ. Identification of four novel mutations in severe methylenetetrahydrofolate reductase deficiency. *Eur J Hum Genet* (1998) 6(3):257-65.
2. Tonetti C, Saudubray JM, Echenne B, Landrieu P, Giraudier S, Zittoun J. Relations between molecular and biological abnormalities in 11 families from siblings affected with methylenetetrahydrofolate reductase deficiency. *Eur J Pediatr* (2003)162(7-8):466-475. doi: 10.1007/s00431-003-1196-9.
3. Al-Shamsi A, Hertecant JL, Souid AK, Al-Jasmi FA. Whole exome sequencing diagnosis of inborn errors of metabolism and other disorders in United Arab Emirates. *Orphanet J Rare Dis* (2016) 11(1):94. doi: 10.1186/s13023-016-0474-3.
