# Supplementary figures and images for: A Novel Homozygous Non-sense Mutation in the Catalytic Domain of MTHFR Causes Severe 5,10-Methylenetetrahydrofolate Reductase Deficiency
Source: Front Neurol. 2019 Apr 24;10:411. doi: 10.3389/fneur.2019.00411 (PMC6491806; doi:10.3389/fneur.2019.00411)

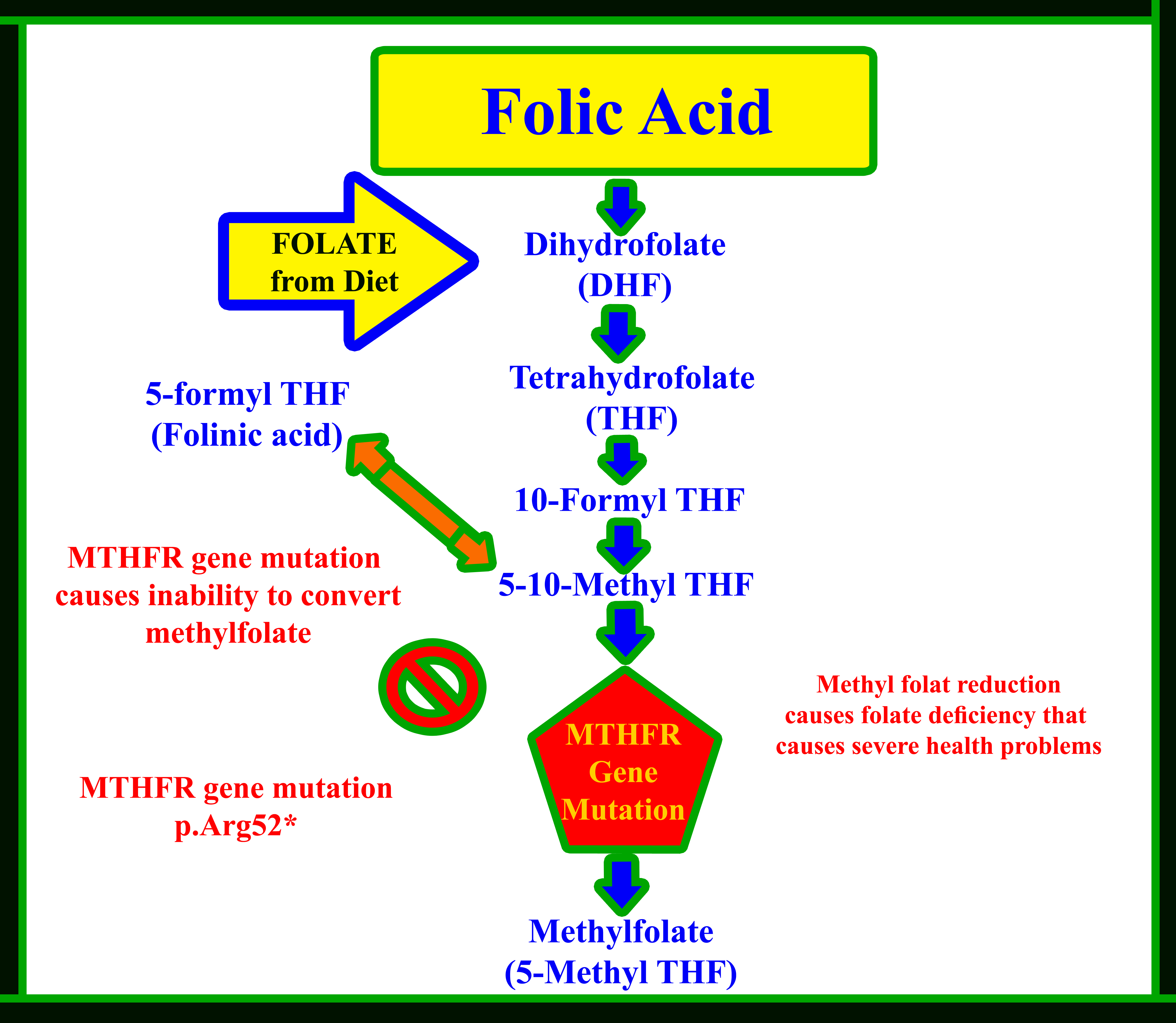

Supplement: Supplementary file 2 [file Image_1.JPEG]
